# Ischemic Gangrene Requiring Right Salpingo-Oophorectomy in a Patient With Ovarian Cyst Torsion and a History of Bilateral Ovarian Cysts: A Case Report

**DOI:** 10.7759/cureus.83595

**Published:** 2025-05-06

**Authors:** Natasha Doshi, Dalya Kanani, Lexi Garber, Ratna H Ramaraju, Anwar S Al-Kunani

**Affiliations:** 1 Gynecological Surgery, Lake Erie College of Osteopathic Medicine, Bradenton, USA; 2 Osteopathic Medicine, Lake Erie College of Osteopathic Medicine, Bradenton, USA; 3 Obstetrics and Gynecology, AMITA Health, Elk Grove Village, USA

**Keywords:** gangrene, ischemia, ovarian torsion, salpingo-oophorectomy, urological emergency

## Abstract

Ovarian torsion is a gynecological emergency that occurs when the ovary gets twisted on its supporting ligaments, which then cuts off the blood supply to the ovary, causing ischemia. This commonly leads to symptoms such as abdominal pain, nausea, and vomiting. Having a history of ovarian cysts can increase the risk of ovarian torsion. In this case report, we will discuss a 22-year-old woman with a history of bilateral ovarian cysts who was diagnosed with gangrenous ovarian torsion. This study will highlight the importance of treating ovarian torsion in a timely fashion in order to prevent undesirable complications, such as gangrene of the ovary, as seen in our patient’s case. Gangrene and other necrotic processes can occur from ovarian torsion due to twisting of the ovary, thus causing a lack of blood supply, which is considered a gynecologic emergency. Once the ovary becomes gangrenous, it is no longer considered viable and treatment options can become limited, as surgical removal is almost always required. Additionally, important topics need to be addressed, such as epidemiology, pathology, and infertility. This is clinically relevant because it raises awareness to diagnose and treat ovarian torsions in a timely manner, to prevent unwanted outcomes, such as gangrenous ovaries or future infertility issues.

## Introduction

Ovarian torsion is a gynecological emergency that occurs when the ligaments that support the adnexa are twisted, causing disruption of blood supply to the ovaries [1]. The infundibulopelvic ligament suspends the ovaries, and it contains ovarian vessels responsible for its blood flow [1]. Ovaries have dual blood flow from uterine and ovarian arteries. When these arteries are twisted and blood flow is disrupted, this causes acute symptoms of severe pain, and it is a true surgical emergency [1]. Complications can lead to necrosis and infertility if treatment is not intervened early. Ovarian masses greater than 5 cm are a major risk factor for ovarian torsion. Studies found that 46% of ovarian torsions were caused by neoplasms, and 48% were caused by ovarian cysts. Eighty percent of these masses were found among women under the age of 50, and 89% of these masses were found to be benign [1]. Therefore, younger, reproductive-aged women are at a higher risk [2]. Torsion occurs when the ovary twists around the axis of the suspensory ligament of the ovary, and first, the venous outflow is disrupted, followed by obstruction of the arterial flow [2]. The right side has an increased space in the pelvis, due to the location of the sigmoid colon being on the left side. As a result, right ovarian torsion is more common [2].

Treatment would include surgical detorsion and an attempt to salvage the ovary, and in 90% of cases of patients who underwent detorsion, ovaries have preserved function following the detorsion surgery [3]. However, if the patient has a gangrenous ovary, such as in our patient’s case, the ovary must be surgically removed [3]. The purpose of this study is to highlight the rarity of developing a gangrenous ovary from an ovarian torsion, which is only an estimated incidence ​of 4.9 per 100,000 [4]. Our patient is only 22 years old but having a gangrenous ovary, which warranted a right salpingo-oophorectomy, affects her future fertility. This uncommon diagnosis warrants a thorough discussion to improve the outcomes regarding women’s health and prevent unwanted gynecological outcomes.

## Case presentation

A 22-year-old white woman (G0) presented to the gynecology clinic for intermittent lower abdominal pain in the right lower quadrant (RLQ). She also reported concerns that her oral contraceptive pills (levonorgestrel and ethinyl estradiol) are contributing to feelings of depression and anxiety. 

Past medical history included a laparotomy and cystectomy for bilateral dermoid cysts at 17. At the age of 21, ultrasound revealed a normal-sized uterus with a thin endometrium and large left dermoid cyst of 8.2 cm x 9.5 cm. A second laparotomy and ovarian cystectomy were done for this. Five months later, postsurgical follow-up was done. Ultrasound revealed that the uterus is of normal size, and ovaries were within normal limits, with no evidence of cysts at the age of 21. The patient visited the emergency department for evaluation of unremitting abdominal pain at the age of 22. Ultrasound revealed that the right ovary had a fluid-filled cyst 57 mm x 31 mm, with the left ovary within normal limits. The patient was informed that her cyst is simple-appearing in nature and not concerning, as its small size, her regular menstrual cycle, and a lack of growth on other abdominal structures suggested a lack of necessity for further evaluation or in need of a surgical consult at this time. 

Two weeks after visiting the emergency department, at the age of 22, the patient presented to the gynecology clinic due to experiencing lower abdominal pain and feelings of depression and anxiety. It was communicated to the patient that she was taking the lowest dose of estrogen for her oral levonorgestrel and ethinyl estradiol contraceptive pills.

**Figure 1:**
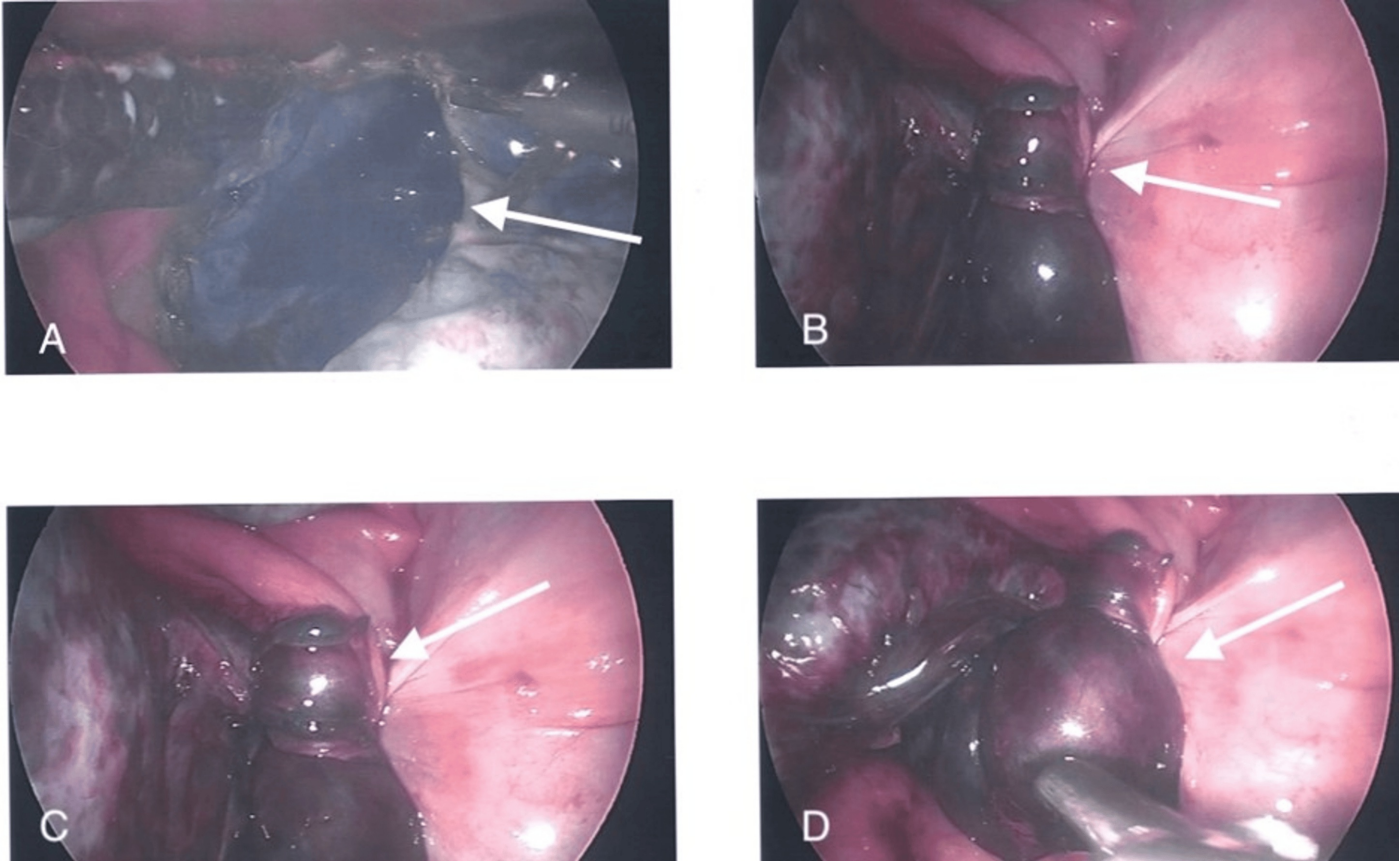
A, B, C, D all demonstrate the gangrenous, necrotic ovary (arrows). At this stage the whole right adnexa was twisted three to four times and completely damaged with gangrene (black and blue tissue), as seen represented by the arrows.

**Figure 2:**
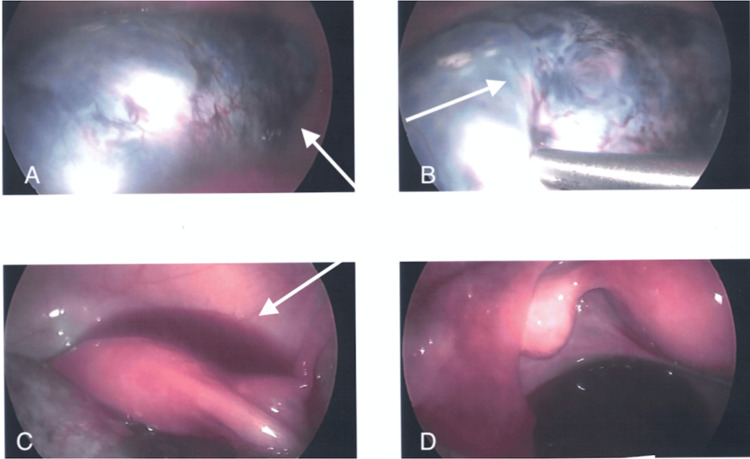
A, B, C, D all demonstrate the ovarian cyst wall, which is extensively hemorrhagic.

Due to the patient’s symptoms on clinical examination and history of ovarian cysts, an ultrasound was done at the gynecology clinic to evaluate for possible ovarian torsion. Ultrasound measures a cyst at 10.6 cm x 9.5 cm x 1.6 cm, and confirmed the presence of an ovarian torsion diagnosed with ultrasound. Surgical intervention was undertaken. During surgery, there was an attempt to de-roof the ovarian cyst and remove the rest of the fluid and untwist the ovary and tube; however, the tissue was not viable. The ovary was completely damaged and with macerated surface and bleeding from multiple points (Figure 1). At this stage, the whole right adnexa was twisted three to four times and completely damaged with gangrene, as discovered with black and blue tissue present. Visualization showed a considerably hemorrhagic ovarian cyst wall (Figure 2). A decision was made to continue with the right salpingo-oophorectomy using the sono scissor. The cyst with the right adnexa was removed in two pieces due to its large size.

After surgical resection, the tissue was sent to pathology for further evaluation. The pathology report noted that the ovarian cyst wall was extensively hemorrhagic. The lining consisted mostly of attenuated epithelium. In a few sections, there was notable keratinizing stratified squamous epithelium as well as underlying adnexal sebaceous glands. Ovarian health was discussed with the patient in order to emphasize the approach taken to preserve ovarian function and fertility as well as create the lowest amount of scarring. The patient was advised to follow up with gynecology as needed.

## Discussion

Epidemiology 

Incidence of ovarian torsion in patients who receive treatment for adnexal masses in a surgical environment ranges from 2 to 15% which makes the occurrence not unusual for women of childbearing age [5]. Likelihood of developing ovarian torsion increases in women of reproductive age [1]. Risk of ovarian torsion increases with masses like tumors and cysts with 80% of cases being patients under the age of 50 [1]. Other risk factors include a history of pelvic surgery, prior pregnancy, and previous fertility treatments [1]. Clinical courses may either have intense nausea and vomiting coupled with an episode of vague, unremitting pain, or cyclic attacks of pain followed by asymptomatic intervals where torsion and detorsion occur [6].

Pathology 

Anatomically, torsion occurs when the ovary twists over the infundibulopelvic ligament or utero-ovarian ligament [1]. The infundibulopelvic ligament contains the ovarian artery and vein, and torsion of this ligament leads to swelling and obstruction of blood flow. The ovarian blood supply primarily comes from the ovarian artery which originates from the abdominal aorta [7]. The ovarian artery goes through the suspensory ligament then goes along the mesovarium and the mesometrium to anastamose with branches of the uterine artery [7]. The ascending branch of the uterine artery supplies blood from both the abdominal aorta and the internal iliac artery [8].

Ovarian torsion serves to twist the infundibulopelvic ligament and the utero-ovarian ligament. Typically, venous outflow is obstructed first, followed by arterial inflow since arteries are thick-walled and less compressible than thin-walled veins; however, once torsion occurs, the collateral flow cannot fully compensate as both arterial inflows are compromised [1]. The patency of these arteries in the setting of blocked outflow leads to ovarian edema, which then exacerbates vascular compression, increases intravascular pressure, and drives a cyclical decline in outflow including blood that could enter through collateral circulation [1]. While collateral flow can offer alternative avenues for blood to flow, it cannot delay ischemia due to mechanical torsion indefinitely considering that the arterial supply of the ovary is predominantly from the larger ovarian artery [8]. Consequently, ischemia, necrosis, and local hemorrhage of the ovary are bound to occur over time without surgical intervention. Infarction can occur in a matter of hours, so detorsion is needed to restore oxygenation via adequate perfusion of the ovary [1]. Additionally, presence of the sigmoid colon in the left pelvis is believed to prevent torsion on that side, with right ovarian torsions being more likely due to a longer utero-ovarian ligament [1]. Gangrene of the ovary is, however, atypical due to the multiple sources of collateral circulation [1]. 

Ovarian cysts and oophorectomy

A woman presenting with abdominal pain in the iliac fossa region, along with vomiting and nausea requires gynecological intervention, especially if they have a history of ovarian cysts. Painful ovarian cyst pain should raise concern of ovarian torsion, and should be treated as a surgical emergency [9]. There are reported cases of successful ovarian detorsion surgery, and in recent years there has been increased utilization of conservative surgery for ovarian torsion to attempt preserving the ovary [1]. Our patient had a right-sided oophorectomy because her ovary was necrotic during surgery. Under other circumstances, performing an oophorectomy during detorsion is justified when there is concern of malignancy. Our patient had a preexisting dermoid cyst, which is a non-functional cyst. It develops from the non-cancerous growth of sebaceous glands, and the sebum produced leads to the formation of cysts. Recent reports have found that dermoid cysts can turn into cancer and become malignant [10].

Infertility 

Women with prior surgical history of ovarian cyst surgery are more likely to report subsequent infertility compared with women with no prior ovarian cyst surgery [11]. A study [12] found that women who underwent a one-sided salpingo-oophorectomy had a 53.9% increased risk of infertility, and younger patients had an improved chance of getting pregnant. Right-sided salpingo-oophorectomy has been shown to have reduced pregnancy outcomes compared to the patients who underwent left-sided salpingo-oophorectomy. Surgical intervention for ovarian torsion leads to concerns regarding the approach to ensure preservation of fertility along with complete resolution of gynecological emergencies in order to ensure proper blood supply to the ovary. Improper management of torsion leading to ischemic damage can lead to loss of function and viability of ovarian follicles and ovarian function must be prioritized.

Gangrenous ovary

Gangrene is a result of ischemic necrosis of tissue due to loss of blood supply [13]. Loss of blood supply and infective processes when an area is not promptly diagnosed as having lost its vascular supply can lead to symptoms such as abdominal pain and swelling along with a mass, as seen in this patient [14]. It can occur due to comorbid conditions such as diabetes mellitus, smoking, atherosclerosis, trauma, or peripheral artery disease [13]. This condition is severe and can appear morphologically as dry, shriveled, blue or black tissue [14]. Gangrene of the ovary can occur as a complication of ovarian torsion such as in this case due to a rapid loss of blood supply [14]. Ischemic necrosis of the ovarian tissue due to the compromised vascular supply can present with abdominal pain and a mass as seen in this patient [15]. Typically, gangrene of the ovary is not seen as the ovary receives a rich blood supply from the ovarian arteries that originate in the abdominal aorta. These ovarian arteries along with ascending branches of the uterine artery create multiple sources of collateral circulation, allowing for substantial perfusion of the ovary [8]. Delay of diagnosis can lead to compromised blood supply and in this case, led to a unique presentation of a gangrenous ovary in the setting of gynecological emergency.

## Conclusions

Ovarian cysts and torsion can be common diagnoses but are considered a gynecological emergency. If not recognized and treated promptly, an ovarian torsion can cause a gangrenous ovary. This severe and rare complication should be kept in mind when diagnosing and managing affected patients. Keeping a vigilant attitude while treating reproductive-aged women who present with acute abdominal pain as in this case can avoid potentially disastrous consequences. This case emphasizes the necessity of early and accurate diagnosis to improve patient outcomes and potentially avoid necrosis and ischemia as well as reinforces considerations to preserve fertility whenever possible. Due to unwanted complications, surgical intervention, including detorsion, and complete removal of the ovary is almost always required if the ovary becomes gangrenous. In cases of severe ovarian necrosis, a complete salpingo-oophorectomy is the definitive treatment. For patients undergoing complete salpingo-oophorectomy, the issue of future infertility is crucial to discuss with affected patients, especially those who are within reproductive age and desire future pregnancy. Future research must emphasize the need to improve diagnostic tools and expand surgical methods to preserve fertility in young patients. Early and accurate diagnosis of gangrenous ovarian torsion is necessary to improve patient reproductive health and women's health outcomes. 
